# Fish welfare in farms: potential, knowledge gaps and other insights from the fair-fish database

**DOI:** 10.3389/fvets.2024.1450087

**Published:** 2024-10-09

**Authors:** Caroline Marques Maia, João Luis Saraiva, Eliane Gonçalves-de-Freitas

**Affiliations:** ^1^FishEthoGroup Association, Faro, Portugal; ^2^Alianima, São Paulo, Brazil; ^3^CAUNESP—Centro de Aquicultura da UNESP, Jaboticabal, Brazil; ^4^fair-fish, Uster, Switzerland; ^5^Centre of Marine Sciences (CCMAR/CIMAR-LA), Faro, Portugal; ^6^Departamento de Ciências Biológicas, Instituto de Biociências, Letras e Ciências Exatas, Universidade Estadual Paulista (UNESP), São José do Rio Preto, São Paulo, Brazil

**Keywords:** aquaculture, farmed fish, fish behavior, reproduction, welfare criteria

## Abstract

An adequate understanding of fish behaviors and their interaction with farm-specific environments is pivotal for enhancing fish welfare in aquaculture. The fair-fish database aims to provide a consistent overview of the welfare of farmed fish. This platform consolidates ethological knowledge into profiles of farmed aquatic species. Its WelfareCheck profiles are organized around welfare indicators, with each criterion receiving classifications (no findings, unclear, low, medium, and high) regarding the likelihood and potential for individuals of a given species to experience good welfare in aquaculture systems, along with the associated certainty level. These criteria include home range, depth range, migration patterns, reproduction, aggregation patterns, aggressive behavior, substrate needs, stress responses, malformations, and slaughtering protocols. We investigated which of these 10 criteria are most relevant to the overall welfare of a species, considering the likelihood, potential, and certainty of good welfare in aquaculture. To achieve this, we reviewed and recorded the high classifications across each criterion and dimension from all published WelfareCheck profiles. To further investigate knowledge gaps across the criteria, we also recorded classifications marked as *unclear* and *no findings*. These were then compared across the criteria to assess the frequency of such classifications. While no significant differences were found between the criteria regarding the likelihood that the surveyed species meet their basic welfare needs, criteria related to reproduction, slaughter practices, and substrate needs demonstrated a high potential for better welfare outcomes. Moreover, reproduction and migration patterns exhibited high certainty in the available literature. Based on these findings, we conclude that improving the reproduction of farmed aquatic species, considering their natural needs and behavior, could be an effective and reliable approach to improving welfare. However, we also found a low certainty of information on aggression and an absence or conflicting data on home range, aggregation patterns, stress, and malformations. This highlights an urgent need for research in these areas, which are fundamental for developing more accurate assessments and recommendations for farmed aquatic species.

## 1 Introduction

The landscape of seafood provision for human consumption has been fundamentally reshaped by aquaculture, which furnished 94.4 million tons of aquatic animals in 2022. This amount represents 51% of the world’s aquatic animal production, surpassing fishing for the first time in history (1). Since 2020, this industry has witnessed an impressive surge, boasting an annual growth rate of 6.6%, leading to 57% of aquatic animal products being directly consumed by humans (1). Projections indicate that this trajectory will continue, with production expected to reach approximately 111 million tons by 2032, which represents a global surge of 17% compared to 2022 (1). Therefore, it is urgent to take into account the welfare conditions of farmed fish, as they are sentient beings capable of feeling pain and suffering (2–7). Furthermore, fish frequently face challenges in aquaculture conditions, including spatial constraints, unnatural groupings, barren surroundings, and an array of artificial stressors (8, 9). These conditions diverge significantly from the natural habitats of farmed aquatic species.

A compounding issue is that, unlike terrestrial counterparts, where food production predominantly centers around approximately 26 species, fish farming involves hundreds of species, each with its own particularities and needs. Approximately 17 staple species represent 60% of global aquaculture production, while other species are mainly important at local levels (1). Thus, despite extensive literature on the biology and welfare of terrestrially farmed animals, significant knowledge gaps remain for a multitude of aquatic farmed species (10). Numerous studies focus on farming, often aiming to optimize growth, fertility, and size in various fish species (11). On the other hand, welfare research is typically limited to a few select species and remains fragmented in the literature.

Three approaches, initially proposed by Fraser et al. (12), are commonly used to evaluate fish welfare in aquaculture: the function-based, feelings-based, and nature-based approaches. The function-based approach considers fish as well if their biological functions are appropriately expressed, that is, fish feed and grow properly, show no signs of distress, and are free of illness. This approach involves measuring hormones (e.g., cortisol), metabolism, growth rate, reproductive performance, and immunological indicators (13). The nature-based approach assumes fish are well when expressing their natural behavioral repertoire. This approach involves behavioral evaluations of aggressive interactions, courtship, feeding, and predator avoidance (14). Finally, the feelings-based approach assumes that fish are sentient beings that experience good welfare when their affective states are positive or at least not negative. This approach requires the evaluation of emotional states, which are more difficult to assess in fish. However, both behavioral (15, 16) and neurophysiological indicators (17, 18) have been used to evaluate fish affective states. Studies have demonstrated that fish are able to experience emotion-like states, displaying behaviors related to fear, stress, anxiety, aggressiveness, and social preferences. They can even learn to avoid negative experiences, such as frightening stimuli (7, 17, 19). Within this context, an overview of ethological knowledge regarding fish welfare is fundamental, as several behaviors emerge as essential indicators of an animal’s physiological and psychological state (11, 16, 17, 20, 21). Ideally, welfare indicators should integrate not only the physical welfare of the fish (22) but also their emotional state (23, 24), in addition to acknowledging their intrinsic needs (8, 25). An expansive scientific comprehension of fish’s natural behaviors and needs, alongside the conditions they encounter on farms, is indispensable for offering well-founded recommendations to improve their welfare in aquaculture. The fair-fish database provides a consistent overview of the welfare of farmed aquatic species worldwide. It is a pioneering open-access platform that systematically categorizes ethological knowledge into species profiles.

### 1.1 The fair-fish database and welfare criteria covered in its WelfareCheck profiles

The fair-fish database serves as a platform where scientific knowledge concerning the behavior, natural needs, and rearing conditions of aquatic species in farms is meticulously organized, classified, critically assessed, and scrutinized (10). Among the available data, the fair-fish database introduces WelfareCheck, a profile providing a quick assessment of the welfare conditions for farmed individuals of each species based on literature-derived information, which is methodically organized into 10 fundamental criteria that rely on welfare indicators, uncovering concerns and potential solutions (10). These criteria encompass measurable variables that are most likely to affect the welfare of aquatic farmed animals, allowing their application across species farmed worldwide (10). The criteria include data on home range, depth range, migratory patterns, reproduction, aggregation behavior, aggressiveness, substrate needs, stress responses, malformation rates, and protocols for farmed fish stunning and slaughtering.

### 1.2 Rationality behind the selection of the welfare criteria for the WelfareChecks

The set of 10 welfare criteria of farmed aquatic individuals composing the criteria of the WelfareChecks within the fair-fish database aims to emphasize the key challenges fish face in farming (10). Fish in aquaculture face several constraints, including limited space, manipulation and handling, low environmental complexity, artificial aggregation patterns, regulated feeding regimes, and the slaughter process. Consequently, the selection of criteria strategically reflects these imposed conditions. Such welfare criteria are based on indicators that can be directly measured (or at least assessed) and relate to home range requirements, depth range utilization, migration pattern/habitat change (spatial constraint), free reproduction (physiological and behavioral constraints), aggregation behaviors (social constraint), aggressive behaviors (behavioral and social constraints), substrate and shelter requirements (environmental and ecological constraints), handling/management stress (physiological and mental constraint), malformation rates (physiological constraint), and stunning/slaughtering protocols (death constraint). Some criteria may use groups of indicators that relate to specific constraints (for example, criteria related to social or ecological constraints may use physiological and behavioral indicators to answer the question). For detailed information about the meaning of these constraints and why they affect fish behavior and welfare, please refer to Saraiva et al. (10).

Furthermore, the primary rationale for selecting these criteria is based on the premise that they should enable a succinct and targeted evaluation of the welfare status of farmed individuals of a given species (10). Thus, the WelfareChecks may be viewed as short profiles organized into 10 criteria designed to encompass the integrative nature of welfare, encompassing its mental, physiological, and natural approaches (12, 26). Each of these criteria receives ratings in the WelfareChecks of the fair-fish database, according to the rationale detailed below.

### 1.3 Rationality behind the ratings given in the criteria of the WelfareChecks

The ratings given in the criteria of each WelfareCheck include *low*, *medium*, *high*, *unclear*, or *no findings* classifications in relation to a given species experiencing a good welfare level in farms. These are given in three distinct dimensions, which are: (1) the likelihood of the individuals of a certain species experiencing a high level of welfare under common farming conditions, (2) the potential of the individuals of a certain species to experience a high level of welfare under improved farming conditions, and (3) the level of certainty about these assessments [(10); https://fair-fish-database.net/]. An *unclear* rating is given when there is conflicting/insufficient information, whereas a *no findings* rating is given when literature-based information is absent. *Low*, *medium,* and *high* ratings are given to indicate a low, medium, or high likelihood, potential, or certainty for a good welfare level, respectively. Thus, a *high* rating reflects a strong likelihood, potential, or certainty that a species experiences a good welfare level in farms. For more details about the rationality behind the classification of these dimensions in each criterion, please see Maia et al. (27). The WelfareScore is computed by summing the frequency of high classifications across the criteria in each of the three dimensions. It serves as a benchmark for assessing and improving the welfare of individuals from aquatic species in aquaculture systems worldwide. A summary of definitions and variables used to apply the WelfareScore is included in Supplementary Table S1.

Each WelfareChecks criterion is segmented by distinct life stages, corresponding to developmental phases in farming environments: eggs, larvae (hatchery), juveniles (nursery), adults (grow-out), and spawners (broodstock) (10). The information from the literature review that is added for each of these life stages for a certain species in its WelfareCheck encompasses knowledge in its natural habitat (i.e., in the “wild”) and under its farming conditions (i.e., on the “farm”), distinguishing the different farming methods when literature is available (10).

To assign ratings (*low*, *medium*, *high*, *unclear,* or *non-existent*) for each dimension (likelihood, potential, and certainty of good welfare) across various criteria, existing knowledge about a species’ behavior in the wild is compared with data from its farming conditions. This comparison helps draw conclusions about the welfare of farmed individuals of the species with respect to each specific criterion. For instance, if the depth range criterion in a species’ WelfareCheck indicates that the species naturally inhabits depths of 2–3 m in the wild but is reported to be farmed both in cages of only 1 m and in ponds of 3 m depth, this results in a low rating for the likelihood and a high rating for the potential dimension of this criterion. This suggests that the farmed individuals of this species have a low likelihood of experiencing good welfare under current farming conditions, as some aquaculture systems (e.g., cages) do not accommodate their full depth range. However, the species has a high potential for good welfare under improved conditions, as certain systems (e.g., ponds) do meet its depth range needs. Conversely, if another species’ WelfareCheck shows that it also naturally lives within the same depth range (2–3 meters) but is farmed in cages of 1 meter and ponds of 2 meters, the ratings would differ. While the likelihood of experiencing good welfare remains low under current farming conditions, the potential for a good welfare level is rated as medium. This occurs because there is some overlap between the wild (2–3 m) and farming (1–2 m) depth ranges for this species, although the entire wild depth range is not fully accommodated.

In practice, the likelihood rating reflects the worst-case scenario for the species under current farming conditions, while the potential rating focuses on the best-case scenario. Although it could be arguable whether potential should be included in welfare assessment, the rationale is that potential is determined by the best available farming conditions in the literature, whereas likelihood is based on the worst cases found in such literature, which are both inferred from comparisons with the wild needs and behaviors of a given species. In other words, the likelihood of farmed individuals of a given species experiencing a high level of welfare on farms is scored considering the worst reported farming conditions compared to the natural behaviors and needs of such species in a given criterion. Similarly, the potential for farmed individuals of a species to experience a high level of welfare on farms is scored based on the best-reported farming conditions compared to the natural behaviors and needs of that species in a given criterion. In this same example, if several wild and farm papers are directly cited to support such information specifically for the focus species, this is reflected in a high rating for certainty about such likelihood and potential for good welfare regarding the depth range criterion. For more details on the ratings given in the criteria of the WelfareChecks (27).

### 1.4 Objective

Although all criteria of WelfareChecks are relevant for the assessment of fish welfare on farms, some may hold greater significance for the overall welfare of farmed individuals from aquatic species based on what is currently known from the literature. In this context, we evaluated which criteria from the WelfareChecks in the fair-fish database play a decisive role in the state of the art of the welfare status of farmed individuals from aquatic species, considering the likelihood, potential, and level of certainty regarding good welfare. Higher likelihood should indicate criteria with a higher chance of good welfare under current farming conditions, whereas higher potential should indicate criteria with a greater chance of future welfare improvement. However, lower likelihood should indicate criteria poorly addressed in farms currently, whereas lower potential should indicate criteria with little chance for future welfare improvement. Higher or lower certainty should indicate the reliability of these findings, with higher certainty reflecting greater confidence in the data. Additionally, we investigated the primary knowledge gaps throughout the criteria. We aimed to identify the criteria that require more research for most species regarding their likelihood, potential, or certainty of a good welfare level in farms. To do this, we focused on detecting criteria with a higher amount of absent or confusing information in the literature across the WelfareCheck profiles.

## 2 Materials and methods

### 2.1 Research strategy

**Figure 1:**
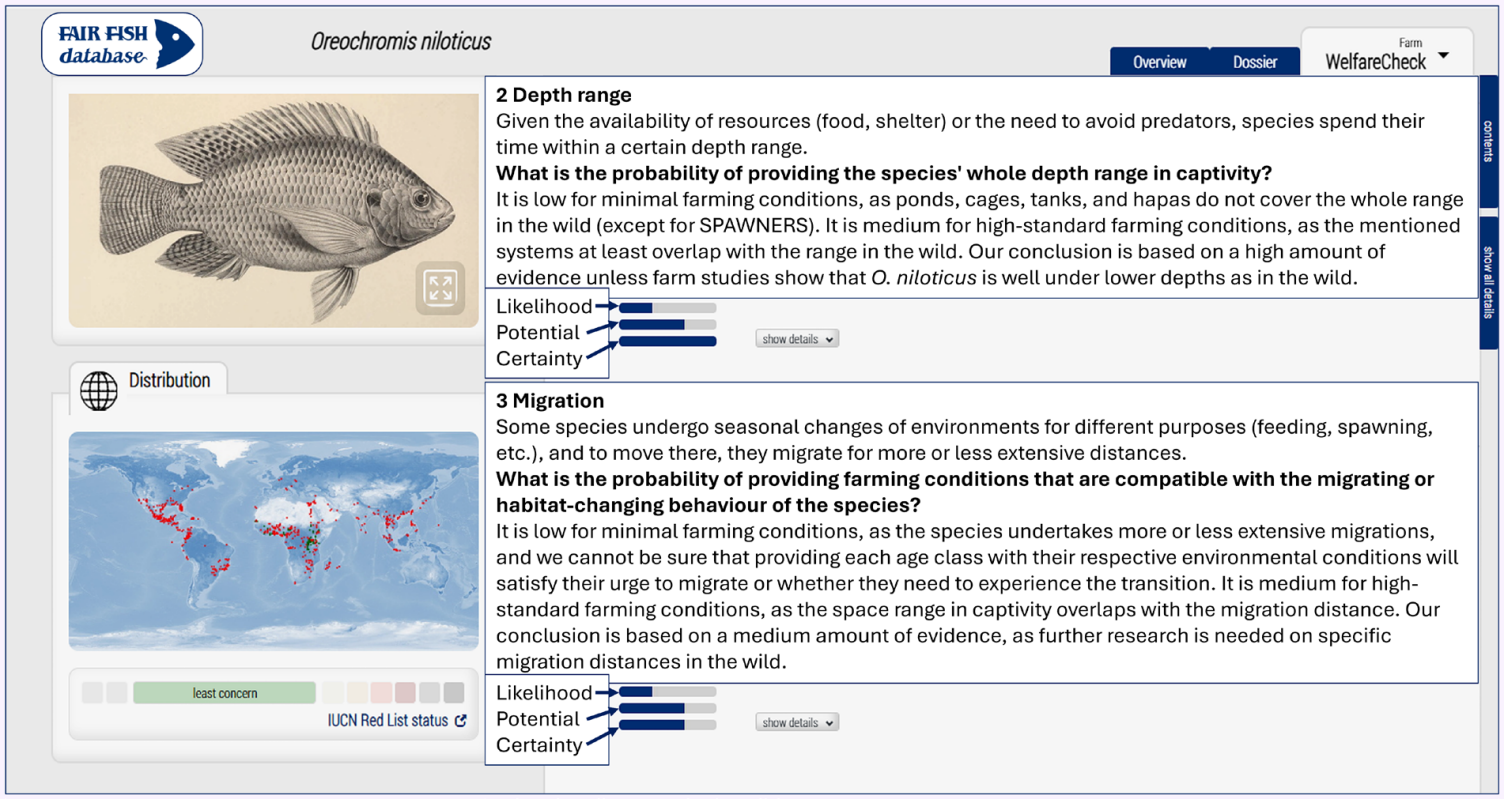
An example of one WelfareCheck, showing classifications in the three dimensions of the WelfareScore (likelihood and potential of experiencing a good level of welfare under farming conditions and the certainty degree about these) for some of the criteria of this profile (depth range and migration). Such classifications include *high* scoring, which is represented by complete blue bars in the image above. *High* classifications were checked and registered for each criterion used in the WelfareChecks independently for each WelfareScore dimension. This is considered to be all WelfareChecks already published in the fair-fish database. The images were printed with permission from the fair-fish database (https://fair-fish-database.net/).

To determine which criteria of the WelfareChecks in the fair-fish database are most influential in the calculation of the WelfareScore, we checked and recorded the high classification for each of the three dimensions, profile by profile (*n* = 83), (Figure 1). Such dimensions include the likelihood and the potential to experience good welfare levels under farming conditions and the degree of certainty about these. This was independently checked and registered for each criterion used in the WelfareChecks (home range, depth range, migration, reproduction, aggregation, aggression, substrate, stress, malformations, and slaughter). As explained above, the possible classifications in each dimension of each criterion are *low*, *medium*, *high*, *unclear*, or *no findings*, but only *high* classifications are considered for the final calculation of the WelfareScore (10). Subsequently, we compared the frequencies of this classification between the 10 criteria to evaluate which ones express the highest frequencies of a high likelihood or potential for good welfare under farming conditions or of a high level of certainty regarding these.

**Figure 2:**
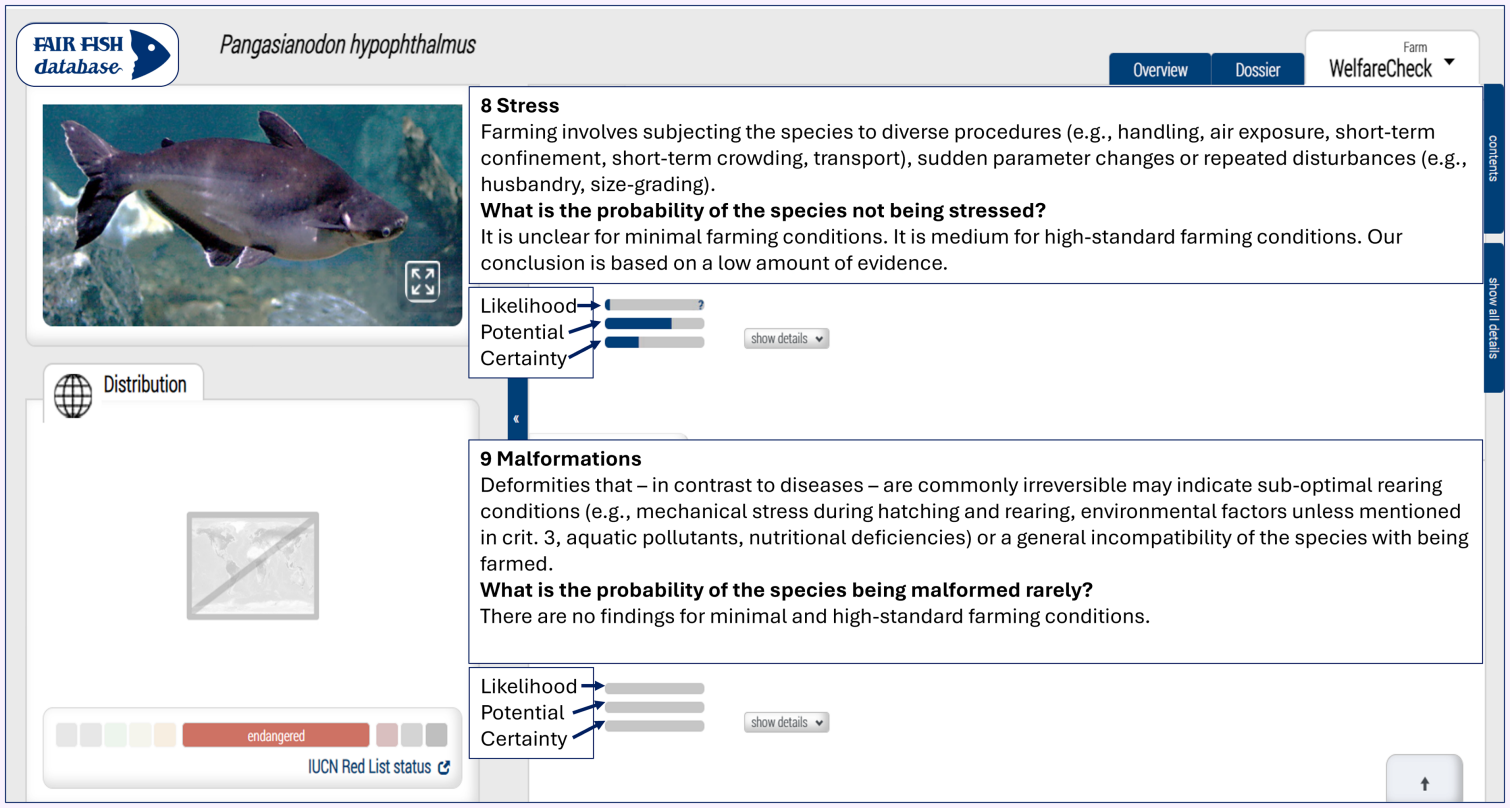
An example of one WelfareCheck, showing classifications in the three dimensions of the WelfareScore (likelihood and potential of experiencing a good level of welfare under farming conditions and the certainty degree about these) for some of the criteria of this profile (stress and malformations). Such classifications include *unclear* and *non-existent* scoring, which are represented by question marks and empty bars in the image above, respectively. *Unclear* and *non-existent* classifications were checked and registered for each criterion used in the WelfareChecks independently for each WelfareScore dimension. This is considered to be all WelfareChecks already published in the fair-fish database. The images were printed with permission from the fair-fish database (https://fair-fish-database.net/).

To better investigate the knowledge gaps throughout the criteria for the farmed individuals from aquatic species with profiles already published, we recorded, profile by profile, the occurrence of classifications as *unclear* and *non-existent knowledge* in each dimension of the WelfareScore for each of the 10 criteria (Figure 2). The frequencies of these occurrences were then independently compared among the 10 criteria for each of the three dimensions, aiming to assess which criteria still have a substantial absence of knowledge or confusing data in the literature.

### 2.2 Data analyses

Taking into consideration each of the 10 criteria used in the WelfareChecks of the fair-fish database, frequencies for the classification as *high* for individuals of species with published profiles were compared between these criteria for each dimension of WelfareScore separately by using the Goodman proportion test [(28), within multinomials]. This analysis was conducted to assess which criteria from the fair-fish database play a crucial role in the welfare state of farmed individuals for WelfareCheck-profiled aquatic species already published on this platform. Furthermore, to evaluate which criteria still express a significant absence of knowledge or confusing data in the literature, the frequencies of classifications as unclear and non-existent knowledge for each of the 10 criteria were also compared among them using the Goodman proportion test [(28), within multinomials] for each dimension of the WelfareScore. For these analyses, the significance level was set at a *p*-value of ≤0.05.

## 3 Results

### 3.1 Welfare criteria: likelihood, potential, and certainty of good welfare for farmed individuals from aquatic species

**Figure 3:**
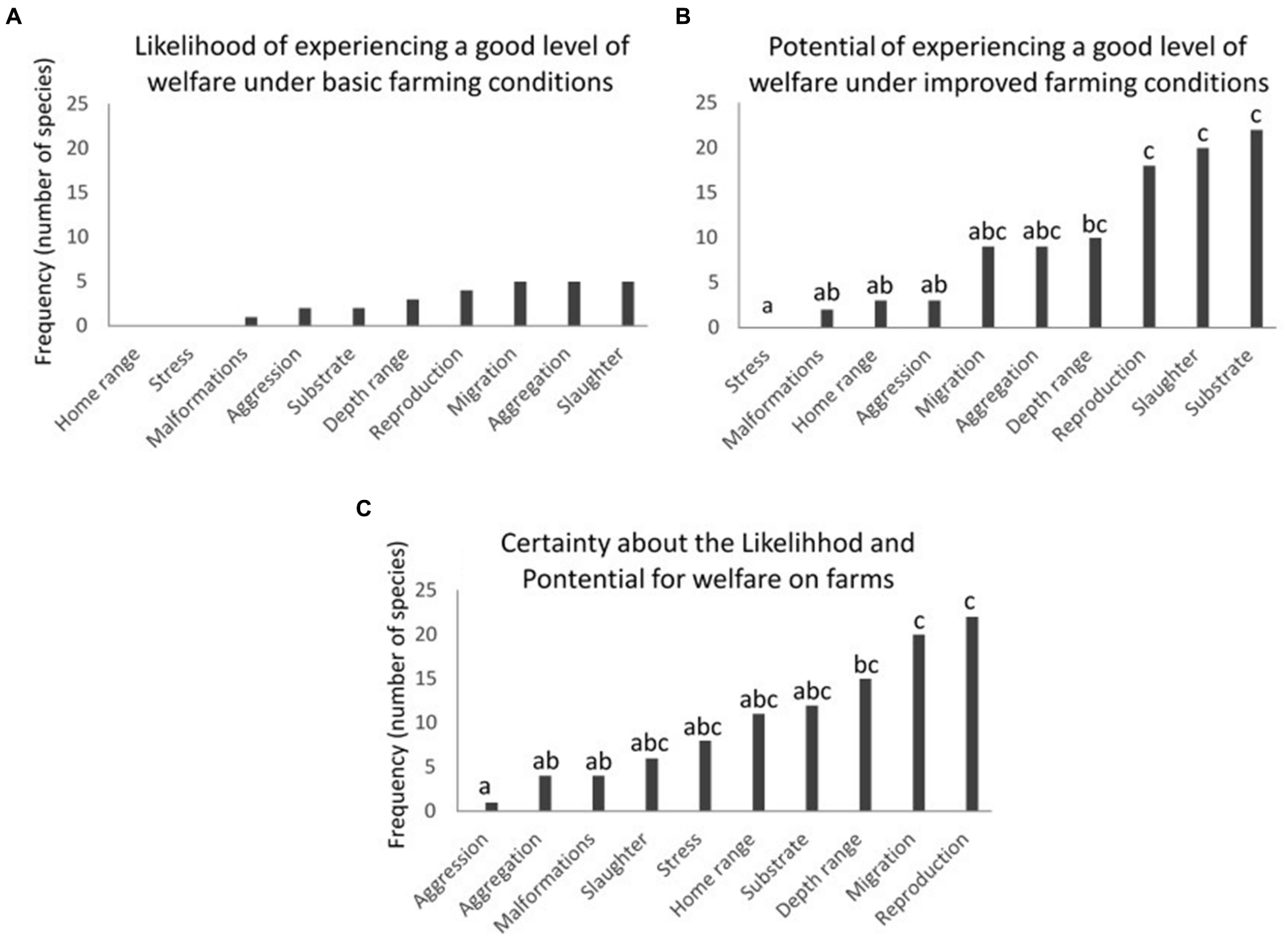
Frequencies of WelfareCheck-profiled farmed aquatic species published in the fair-fish database (*n* = 83), with **(A)** a high likelihood of individuals experiencing good welfare under basic farming conditions or **(B)** a high potential of individuals experiencing good welfare in improved aquaculture systems, or **(C)** a high level of certainty about such likelihood and potential, considering the 10 criteria used in the construction of these WelfareChecks. Different lowercase letters indicate significant differences [Goodman’s proportion test (28)—within multinomials, *p* < 0.05].

Considering the 10 criteria used in the construction of the WelfareCheck profiles in the fair-fish database, there was no significant difference in the frequencies of farmed aquatic species profiles among these criteria regarding the high likelihood of experiencing good welfare under basic farming conditions [Figure 3A; Goodman’s proportion test (28), within multinomials, *p* > 0.05]. The frequencies of profiles with such likelihood were low in all criteria, ranging from 0% (criteria: home range and stress) to 6.2% (criteria: migration, aggregation, and slaughter) (Figure 3A). However, regarding the high potential for experiencing good welfare under improved farming conditions and the high level of certainty about the likelihood and potential for good welfare on farms, there was a significant difference in the frequencies of farmed aquatic species profiles among the 10 criteria [Figures 3B,C; Goodman’s proportion test (28), within multinomials, *p* < 0.05].

The stress criterion for farmed fish showed the lowest frequency of species profiles (0%) with a high potential for good welfare under improved farming conditions (Figure 3B). In contrast, the reproduction (21.7%), slaughter (24.1%), and substrate (26.5%) criteria had the highest frequencies of species profiles with good potential for better welfare under improved farming conditions (Figure 3B). Moreover, the criterion evaluating aggressive responses of individuals presented the lowest significant frequency of species profiles (1.2%) with a high level of certainty about the likelihood and potential for experiencing good welfare in farm conditions (Figure 3C). On the other hand, criteria related to migration (24.1%) and reproduction (26.5%) were those that presented the highest significant frequencies of species profiles with a high level of certainty about the likelihood and potential for good welfare in aquaculture systems (Figure 3C).

### 3.2 Welfare criteria: unclear or non-existent knowledge of the likelihood, potential, and certainty of good welfare for farmed individuals from aquatic species

**Figure 4:**
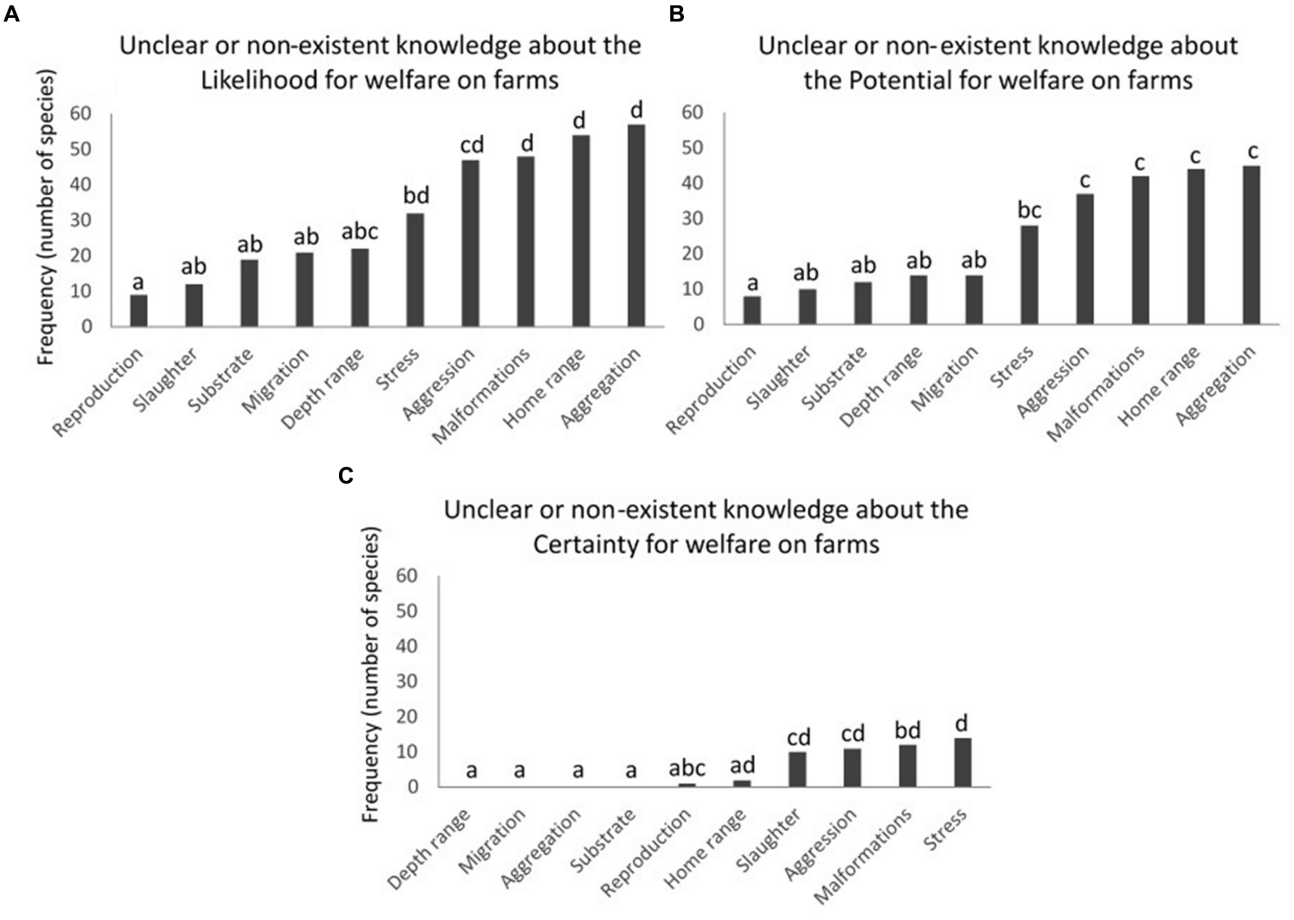
Frequencies of WelfareCheck-profiled farmed aquatic species published in the fair-fish database (*n* = 83) with **(A)** confused or non-existent knowledge about the likelihood of individuals experiencing good welfare under basic farming conditions, or **(B)** confused or non-existent knowledge about the potential of individuals to experience good welfare in improved aquaculture systems, or **(C)** confused or non-existent knowledge about the level of certainty of such likelihood and potential, considering the 10 criteria used in the construction of these WelfareChecks. Different lowercase letters indicate significant differences [Goodman’s proportion test (28)—within multinomials, *p* < 0.05].

There was a significant difference in the frequencies of species profiles among the 10 criteria used in the construction of WelfareChecks in the fair-fish database in all three dimensions of the WelfareScore [Figure 4; Goodman’s proportion test (28), within multinomials, *p* < 0.05]. Significant differences were found among these criteria in the likelihood of experiencing good welfare under basic farming conditions (Figure 4A), the potential for improved welfare in aquaculture systems (Figure 4B), and the level of certainty regarding these findings (Figure 4C).

Considering the likelihood of experiencing good welfare under basic farming conditions (Figure 4A), reproduction was the criterion that presented the lowest significant frequency of species profiles with unclear or non-existent knowledge (10.8%). The same response occurred in relation to the potential to experience a good level of welfare under improved farming conditions (9.6%). However, criteria related to malformations, home range, and aggregation patterns presented the highest frequencies of species profiles with unclear or non-existent knowledge (57.8, 65, and 68.7%, respectively) regarding the likelihood of good welfare under basic farming conditions (Figure 4A). A similar response was also observed for the potential to experience good welfare under improved farming conditions (Figure 4B; malformations = 50.6%, home range = 53%, and aggregation = 54.2%). The aggression criterion also showed a significantly high rate of unclear or non-existent knowledge about the potential for good welfare in improved farming conditions (Figure 4B; aggression = 44.6%).

Regarding the unclear or non-existent knowledge about the certainty level of likelihood and potential for good welfare in aquaculture systems, the criteria on depth range, migration, aggregation, and substrate showed no frequency of species profiles (Figure 4C). In contrast, the criterion related to stress exhibited the highest significant frequency of species profiles with unclear or non-existent knowledge about the certainty of good welfare in farming systems, accounting for 16.9%.

## 4 Discussion

This study investigated the likelihood, potential, certainty levels, and knowledge gaps of the welfare criteria presented in 83 revised profiles of worldwide farmed aquatic species published in the fair-fish database. These criteria include data on home range, depth range, migration, reproduction, aggregation, aggression, substrate, stress, malformations, and slaughter practices. From these, we show that reproduction is the most promising way to reach better welfare conditions for farmed individuals from aquatic species. We also show that future research on aggressiveness, malformations, aggregation, and home range in the welfare context of aquaculture species is needed due to the current uncertainty, absence, or confusion in the available information.

### 4.1 Welfare criteria: likelihood, potential, and certainty of good welfare for farmed individuals from aquatic species

The low frequencies of species profiles with a high likelihood emphasize the very limited chance that, in general, farmed individuals of aquatic species have of experiencing good welfare under basic farming conditions. This has already been demonstrated by Saraiva et al. (10), investigating the 41 WelfareChecks published up to that time, and, more recently, by Maia et al. (27). In this study, we demonstrate that this finding is consistent across all evaluated welfare criteria (e.g., home range, depth, migration, and so on) in the species with WelfareChecks published in the fair-fish database, reinforcing the poor overall welfare state of farmed aquatic species.

The stress criterion presented the lowest frequency of species profiles with a high potential for a good welfare level in farms, thus indicating that the stress response of individuals from aquatic species on farms is hardly mitigated if the conditions of these animals are improved. It is worth noting that the criterion about the stress of the WelfareChecks focuses on handling and transportation issues, which are quite common events in aquaculture. Handling is considered aquaculture’s most direct acute and chronic stressor (10). It is one of the most invasive actions imposed on farmed aquatic animals, significantly impairing their welfare (29).

Nevertheless, reproduction, substrate, and slaughter criteria showed the highest frequencies of species profiles with high potential, which means that there is a greater potential to improve the welfare of farmed individuals from aquatic species regarding their breeding conditions, substrate provision, and humane slaughter compared to other criteria. In this scenario, in the pursuit of improving already operating aquaculture systems in terms of animal welfare, modifications related to reproduction, substrate, and slaughter are more likely to be effective and applicable in practice. Indeed, improved reproduction without manipulation is generally acknowledged as a sign of good fish conditions (30), and humane slaughter is considered a fundamental feature of the welfare of these animals (10). Furthermore, it is clear that the addition of appropriate substrates in the farm conditions as environmental enrichments can help improve the welfare of farmed individuals from aquatic species, especially of the ones that interact with and use substrates in nature (31).

Regarding certainty levels about the likelihood and the potential for a good welfare level under farming conditions, while the criterion about aggressiveness was the one with the lowest frequency of aquatic species profiles with high ratings, criteria about migration and reproduction showed the highest frequencies of such species profiles. These findings highlight the need for further studies on the aggression of farmed aquatic species to increase the level of certainty about it. The production of individuals from aggressive species or the facilitation of aggression due to confinement, density, or aquaculture method is not advisable as it causes injuries, stress, reduced production, and welfare issues (8). Therefore, a higher level of certainty regarding aggression in farmed species will improve the prevention and management of these issues. For instance, Zhang et al. (32) demonstrated that proper social enrichment decreases intraspecies aggression in black rockfish (*Sebastes schlegelii*) and fat greenling (*Hexagrammos otakii*), which are territorial fish species. Furthermore, based on the existing knowledge in the literature, it is evident that, in general, the findings regarding migration and reproduction are more consistent and robust, leading to a higher level of certainty concerning the good welfare of farmed individuals from aquatic species.

### 4.2 Welfare criteria: unclear or non-existent knowledge of the likelihood, potential, and certainty of good welfare for farmed individuals from aquatic species

Because the reproduction criterion expressed the lowest frequencies of species profiles with unclear or non-existent knowledge, considering both the likelihood and the potential for good welfare in farms, this suggests that, in general, there is clear knowledge in the literature about the reproduction of farmed aquatic species. Therefore, such knowledge certainly allows for a better assessment of this criterion regarding the likelihood and potential of the farmed individuals from aquatic species experiencing a good welfare level in aquaculture systems. This is logical, as studies on reproduction are critical not only for aquaculture but also for rehabilitation and conservation programs for aquatic species (33), making them more prevalent.

On the other hand, there is a need for future studies to investigate both the natural needs and farm-related conditions of aquatic species, considering the malformations, home ranges, and aggregation patterns of individuals to better assess their current conditions and potentials for good welfare in aquaculture, as such criteria presented the highest frequencies of species profiles with unclear or non-existent knowledge. Welfare is often impaired by deformities or malformations caused by human action (34). Additionally, farm production involves confining individuals in areas with highly variable dimensions (10), which affects their natural home ranges and densities, further exacerbating welfare concerns. Additionally, because there are many ways to calculate and define fish density in captivity, with the concept itself being complex (35–37), the need to further investigate the welfare impact related to the aggregation patterns on farms is clear.

Furthermore, despite well-known effects of aggressive behavior on fish welfare in experimental studies [e.g. (38, 39),] and ways to deal with this problem [e.g. (40–42),], our findings indicate that knowing whether it is possible to effectively improve aggression problems in aquaculture is still a bottleneck in the current knowledge. The aggression criterion showed a high frequency of species profiles with unclear or non-existent knowledge about the potential for good welfare on farms. Thus, considering that aggression may cause injuries, stress, reduced production (8, 43), and can even impair nutrition (44), growth (45), and the immune system (46), it is crucial that future research better investigate the patterns of aggression in ponds and other rearing systems of farmed individuals from aquatic species.

Regarding the unclear or non-existent knowledge in relation to the certainty of likelihood and potential for good welfare in farms, our findings indicate that depth range, migration, aggregation, and substrate are welfare criteria of farmed individuals from aquatic species with generally existent and not confusing information in the literature, even though the certainty about them may still be low. Such criteria presented zero species profiles with unclear or non-existent knowledge about certainty for good welfare in farms. On the contrary, the stress response of farmed individuals from aquatic species is still uncertain, as this criterion showed the highest frequency of species profiles. This is alarming given that acute and chronic stress from the manipulation and handling of fish, which are the most addressed stressors in this criterion of the WelfareChecks, are the most direct stressors in aquaculture (10). In other words, information about the most invasive actions imposed on farmed aquatic animals and those that most impair their welfare (29) is still confused or non-existent in the literature for a significant portion of farmed individuals from aquatic species with profiles in the fair-fish database.

## 5 Conclusion

Currently, the best opportunities for achieving a high level of welfare for aquatic species in aquaculture lie in improving their breeding conditions, the slaughtering process, and substrate availability, which often exhibit a high potential for good welfare across species profiles. Reproduction, in particular, is well-researched, and often demonstrates a high level of certainty, with the lowest frequency of species profiles showing unclear or non-existent knowledge.
