# Supplementary material for: Fish welfare in farms: potential, knowledge gaps and other insights from the fair-fish database
Source: Front Vet Sci. 2024 Oct 9;11:1450087. doi: 10.3389/fvets.2024.1450087 (PMC11496955; doi:10.3389/fvets.2024.1450087)
Supplement: Supplementary file 1 [file Table_1.pdf]

Table S1. Terms and definitions of variables used to reach a score for fish welfare on fair-fish database.

Terms	Definitions	Details	
WelfareCheck	The set of information depicted from the literature regarding the aspects in aquaculture most likely to affect the welfare of individuals from aquatic species.		
Criteria	Aspects uncovering pivotal concerns and potential solutions on fish welfare. A set of 10 criteria is considered to the welfare score.	1. home range<br>2. depth range<br>3. migration<br>4. reproduction<br>5. aggregation	6. aggression<br>7. substrate<br>8. stress<br>9. malformations<br>10. slaughter
Dimensions	Current conditions, potential for improvement and certainty level about experiencing good welfare under farming conditions.	- Likelihood<br>- Potential<br>- Certainty	
Ratings	Classification inside of each dimension according to available information in the literature.	- High<br>- Medium<br>- Low	These three ratings indicate high, medium or low likelihood, potential or certainty for good welfare, respectively.
		- Unclear<br>- No findings	These two ratings are given when the available information is conflicting or unavailable.
WelfareScore	The sum of the frequencies of high classifications across criteria in each dimension.		
